# Supplementary material for: MetaRibo-Seq measures translation in microbiomes
Source: Nat Commun. 2020 Jun 29;11:3268. doi: 10.1038/s41467-020-17081-z (PMC7324362; doi:10.1038/s41467-020-17081-z)
Supplement: Supplementary file 10 — Supplementary Data 7 [file 41467_2020_17081_MOESM10_ESM.zip › File2/Confidence_VeryHigh_Taxonomy/185701_out.krona.html]

Javascript must be enabled to view this page.

members
magnitude
magnitudeUnassigned
count
unassigned
taxon
rank

185701\_out

4

2
superkingdom
4

4
1239
phylum

4
class
186801

4
186802
order

species
1898207

SRS020394\_contig\_number\_contig-100\_22750.22751SRS077392\_contig\_number\_42451SRS893230\_contig\_number\_1083SRS893366\_contig\_number\_27575
4
